# Peripheral Bromination for Strongly Affecting the Structural, Electronic, and Catalytic Properties of Cobalt Corroles

**DOI:** 10.1021/acs.inorgchem.5c01310

**Published:** 2025-05-28

**Authors:** Sachin Kumar, Arik Raslin, Sruti Mondal, Amir Mizrahi, Natalia Fridman, Atif Mahammed, Zeev Gross

**Affiliations:** † Schulich Faculty of Chemistry, 26747 Technion - Israel Institute of Technology, Haifa 32000, Israel; ‡ Department of Chemistry, Nuclear Research Centre-Negev, Beer Sheva 9001, Israel

## Abstract

The feasibility of a hydrogen-based economy critically depends on the development of catalysts for the hydrogen evolution reaction (HER) that do not rely on Pt or other noble metals. Contemporary efforts are focused on developing first-row transition metal complexes that will be operative at low overpotentials and catalyze the reaction with high efficacy and turnover rates. We now report a surprisingly strong effect of bromide substituents on the structure, coordination chemistry, electronic spectrum, reduction potentials, and catalytic activity of an already electron-poor cobalt corrole. The six-coordinate cobalt­(III) complex of the brominated corrole displays a very nonplanar macrocycle, its axial pyridines are perpendicular to each other, the maxima in the electronic spectrum are red-shifted by almost 70 nm, and it is reduced by 600 mV less negative potentials. It catalyzes the HER from an organic acid in an organic solvent with a very low onset potential of −0.96 V vs. the Fc^+^/Fc couple and has been used for the preparation of a catalyst-modified cathode to produce hydrogen gas from acidic water with 97% Faradaic efficacy at potentials as low as −0.4 V vs. RHE.

## Graphical abstract

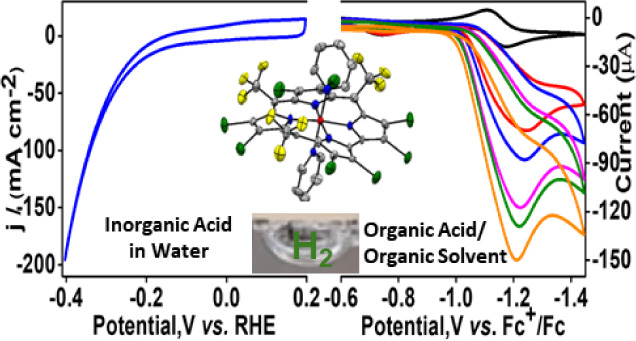

## Introduction

The rapidly growing world population leads to a constantly increasing energy demand, which is mainly generated from fossil fuels, whose burning has a large impact on the greenhouse effect. Hydrogen gas could be a perfect alternative fuel since its burning produces only water,^1,2^ but 95% of its production relies on heavily polluting processes.^3^ Water electrolysis (i.e.,water splitting to its elements) by using renewable electricity is of large interest and need, but the best-performing catalysts for the hydrogen evolution reaction (HER) occurring on the cathode are based on platinum, which is rare and expensive.^4–6^ On the other hand, the natural process is catalyzed by hydrogenases that utilize earth-abundant metals like iron and nickel (Fe/Ni).^6–8^ The quest for achieving efficient HER catalysis by synthetic first-row transition metal complexes requires that they must be of large hydrolytic stability and easily modified for preparing optimal catalysts, with regard to the many variables that affect performance. Metal complexes of porphyrins and similar N4 macrocycles remain at the forefront of this kind of research because their fundamental properties can be fine-tuned through established synthetic manipulations, which further allows for the design of catalysts with specific moieties for boosting activity, selectivity, and even changes in reaction mechanisms.^9–15^

The advantages of corroles relative to porphyrins for reduction catalysis are that they are about 10% smaller and have a trianionic N4 coordination core, consequently leading to exceptional hydrolytic stability of the corresponding metal complexes and enhanced reactivity of their low oxidation states.^16–18^ These properties could potentially enhance electrocatalytic HER activity, but the more negative potential required for the reduction of metal ions chelated by corroles compared to other coordination environments often results in larger overpotentials.^19,20^ Results obtained with second-generation cobalt corroles, with electron-poor aryls on the three *meso*-C positions, revealed that HER catalysis is much improved via halogenation of the β-pyrrole positions.^21–24^ Bromination of the eight β-pyrrole positions of porphyrins has long been known to strongly affect both structural and redox properties of the corresponding metal complexes, which become less planar and more reducible.^25^ However, comparison between cobalt complexes of tetraarylporphyrins and triarylcorroles reveals that bromination of the latter usually does not induce significant structural changes, while the redox potentials are much more strongly affected.^26^

Contemporary research activity focuses on third-generation corroles,^27^ with much smaller *meso*-C substituents, not at least because they are adsorbed much strongerly onto porous carbons that are used for preparing catalyst-modified cathodes.^28^ The current investigation focused on the bis-pyridine cobalt­(III) complex of a third-generation corrole (**1** in Figure 1a), whose *meso-*CF_3_ groups shift the Co­(II)/Co­(I) redox potentials more than aryls like C_6_F_5_ present in the previously studied complexes.^19,29^ Conversion of 5,10,15-tris­(trifluoromethyl)­corrolato cobalt­(III) (bis)­pyridine (**1**) to its octa-brominated analogue, 2,3,7,8,12,13,17,18-octabromo, 5,10,15-tris­(trifluoromethyl)­corrolato cobalt­(III) (bis)­pyridine (**2**), induced a multitude of structural, spectroscopic, and electronic changes: very significant nonplanarity of the macrocycle, perpendicular rather than parallel axial ligands, strongly red-shifted absorption bands, and large differences in the Co^III/II^ and Co^II/I^ redox potentials. In addition, **2** is uncovered as a very efficient catalyst for the electrochemical HER, under both homogeneous (organic solvent and organic acid) and heterogeneous (acidic water) conditions, displaying record values in terms of low overpotential and durability.

**Figure 1:**
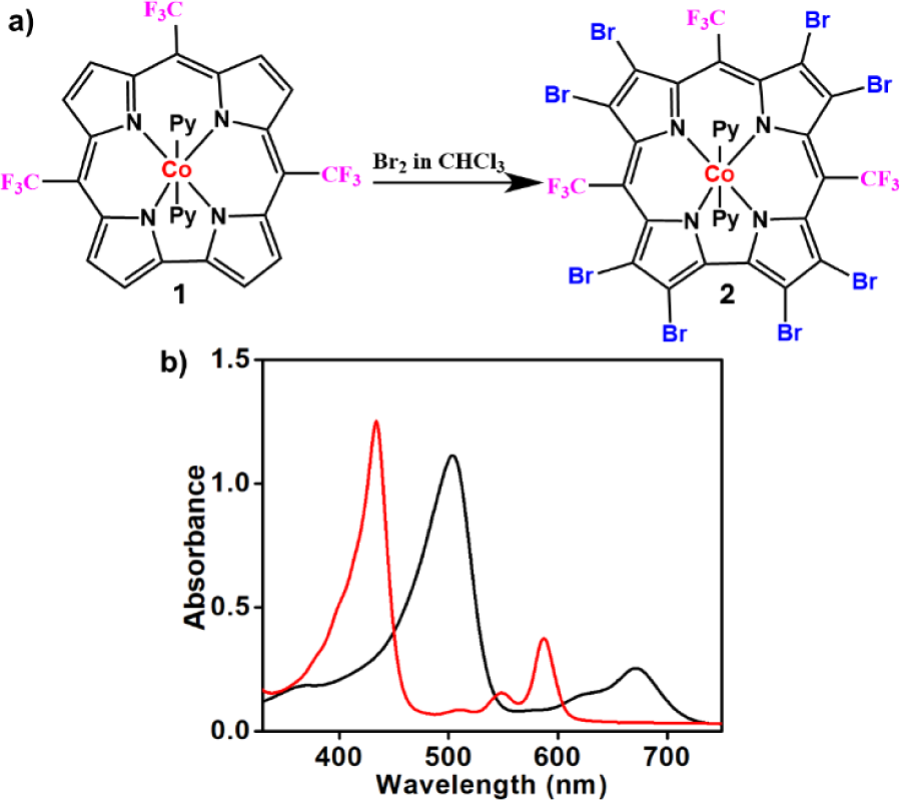
(a) Synthesis of complex **2** via bromination of **1** and (b) electronic spectra of **1** (22.4 μM, red) and **2** (18 μM, black) in dichloromethane.

## Results and Discussion

### Synthesis and Characterization of the Brominated Cobalt­(III) Corrole

The brominated complex **2** was synthesized from **1** (Figure 1a) and first characterized by high-resolution mass spectrometry (HRMS, Figure S5). Consistent with the lack of any H atom in this corrole, only the strongly high-field-shifted resonances of axially ligated pyridine moieties were observed in its ^1^H NMR spectrum (Figures S3). Comparison of the electronic spectra of **1** and **2** uncovers that the most intense bands of the latter are red-shifted by 67 nm relative to **1** (Figure 1b). This is in sharp contrast to the 10–20 nm shift effect of β-pyrrole bromination reported for second-generation metallocorroles,^26a‑26c^ and even exceeds the 40–50 nm shifts characteristic of brominated metalloporphyrins.^26^ Common wisdom attributes the differences between corroles and porphyrins to structural effects, as H/Br substitution at the β-pyrrole positions of the former *usually* induces much less distortion of the macrocycle than for the latter.^26^ This in turn suggests that complex **2** has a heavily distorted macrocycle, likely much more than the cobalt­(III) complexes of both the nonbrominated and brominated *meso*-C_6_F_5_-and other triaryl-substituted corroles, **3**, **4**, **5,** and **6,** respectively (Table 1 and S3).^30–32^

**Table 1:**
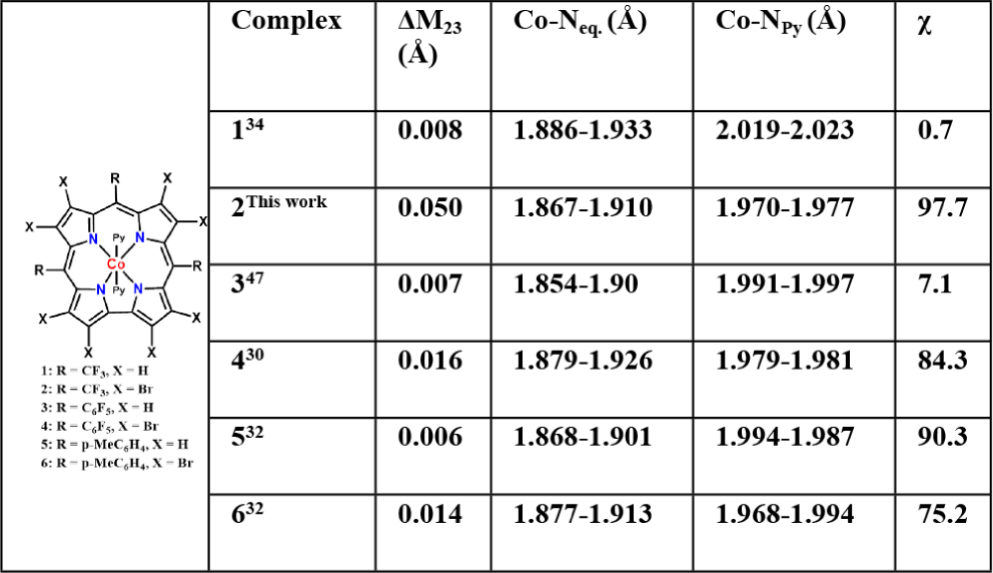
X-Ray Crystallographic Data of (Bis-Pyridine)­Cobalt­(III) Complexes of Brominated and Nonbrominated Corroles^a^

a Δ*M*_23_: deviation of the cobalt ion from the mean plane defined by the 23 atoms; Co–N_eq_ and Co–N_Py_: the average equatorial and axial Co–N bond lengths, respectively; χ: torsional angle between the two axial pyridines.

Analysis of X-ray quality single crystals of **2** (obtained from *n*-hexane/CH_2_Cl_2_/pyridine solution) provides the opportunity to compare the effect of bromination on the cobalt complexes of corroles with *meso*-CF_3_ and *meso*-C_6_F_5_ groups (i.e., **2** vs. **1** relative to **3** vs. **4**).^30,33,34^ The results summarized in Table 1 reveal that the axial Co–N­(pyridine) bond lengths shorten upon macrocycle bromination for both the CF_3_ and C_6_F_5_ substituted corroles but much more so in the former case: by 0.031–0.065 and 0.014–0.015 Å, respectively. In addition, the Co–N­(pyrrole) bonds are shorter in **2** than in **1**, but longer in **4** compared to **3**. A common phenomenon is that the axially ligated pyridine moieties are perpendicular to each other in brominated complexes **2** and **4**, while they are parallel to each other in **3** and **1**. This is reminiscent of studies on cobalt and iron porphyrins, where a correlation between macrocycle ruffling and the nonparallel orientation of axial ligands was established.^35–37^ We note, however, that this phenomenon is present in other cases as well. These include the 5,10,15-tris-*p*-tolyl cobalt­(III) corrole with a quite planar macrocycle and the octabrominated derivative thereof (Table 1: complexes **5** and **6**, respectively) and the very electron-poor 5,10,15-tris-cyano cobalt­(III) corrole that has the combination of perpendicular pyridines and a nonplanar macrocycle.^38^ The angular deviations of the macrocycle due to saddling and ruffling contributions^30,34^ for **2** and the other complexes are provided in Table S3, but the easiest appreciation is provided in Figure 2b,c: while **1** has quite a planar skeleton, **2** exhibits a very high degree of distortion. In terms of the average mean plane deviation of all 23 atoms, it is 0.05 Å for **2** but is only 0.016 Å for **4**.

**Figure 2:**
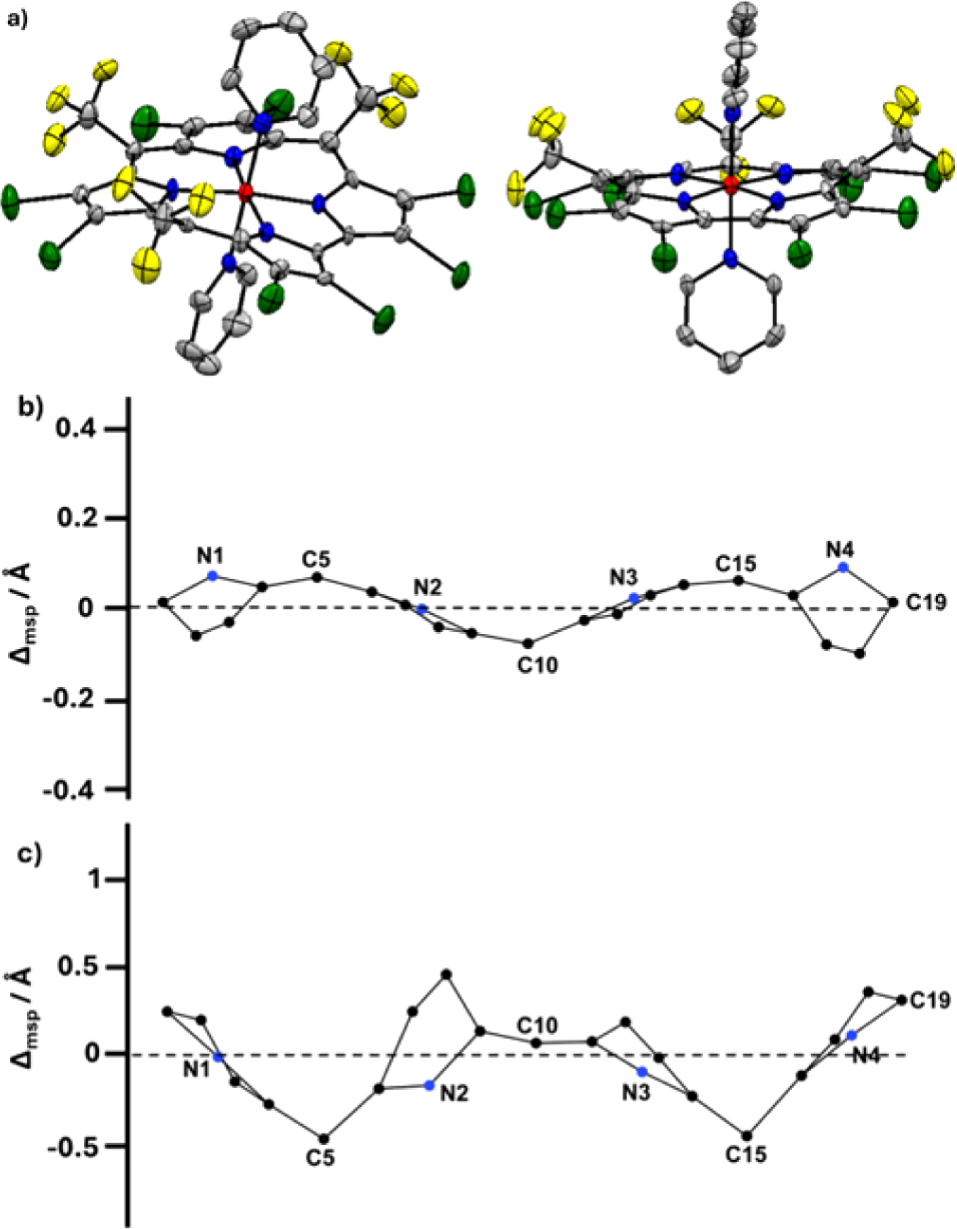
(a) Top and side views of the X-ray structure of **2** (color legend: yellow – fluorine, blue – nitrogen, gray – carbon, red – cobalt, green – bromide) and out-of-plane displacement of the core atoms of (b) **1** and (c) **2** from the least-squares plane of the corrole core. The horizontal axis represents the bond connectivity between atoms.

### Electrochemical Properties of the Cobalt­(III) Corroles

The redox properties of the cobalt­(III) corroles were determined by cyclic voltammetry (CV) in N_2_-purged acetonitrile solutions containing 0.1 M tetrabutylammonium hexafluorophosphate, using an Ag/AgCl reference electrode and a Pt wire as counter electrode, with all potentials relative to the ferrocene/ferrocenium couple. Reductions at the more negative potentials, representing the Co^II/I^ redox couples,^19^ were reversible, with half-wave potentials (*E*_1/2_) of −1.14 V for **2** and −1.67 V for **1** (Figure 3a). The earlier reduction peaks differ even more, −0.74 V for **2** and −1.38 V for **1**, and this Co^III^/Co^II^ transformation proceeds by the “EC” mechanism, which is known to include axial ligand dissociation/association.^39–41^ But there was also a difference in their reoxidation waves: only one very remote (at −0.3 V) for **1** but two (at −0.53 and −0.21 V) for **2** (a′ and a″ in Figure 3a). Based on many previous investigations,^42^ the processes occurring at more and less negative potentials reflect the oxidation of the pyridine-free 4-coordinate and the bis-pyridine 6-coordinate cobalt­(II) complexes, respectively. The fact that both processes are obvious for complex **2** but not for **1** testifies to the larger Lewis acidity of the cobalt ion chelated by the more electron-poor brominated corrole. Supporting evidence of metal-centered reduction processes for both cobalt­(III) corroles was obtained by spectroelectrochemistry (Figure S7). Applying a constant potential of −0.6 V (vs Ag/AgCl) to solutions of **2** leads to very large blue shifts of both the Soret and Q bands, from 503 to 463 nm and from 673 to 629 nm, respectively. These spectral changes are reversible, as reoxidation of the singly reduced species of **2** at a controlled potential of 0.6 V restores the initial UV–vis spectrum completely. Reduction to a potential of −1.1 V leads to less pronounced changes, which are also reversible. These data are similar to observations with other cobalt corrole,^19^ consistent with metal-centered Co^III^/Co^II^ and Co^II^/Co^I^ redox processes.

**Figure 3:**
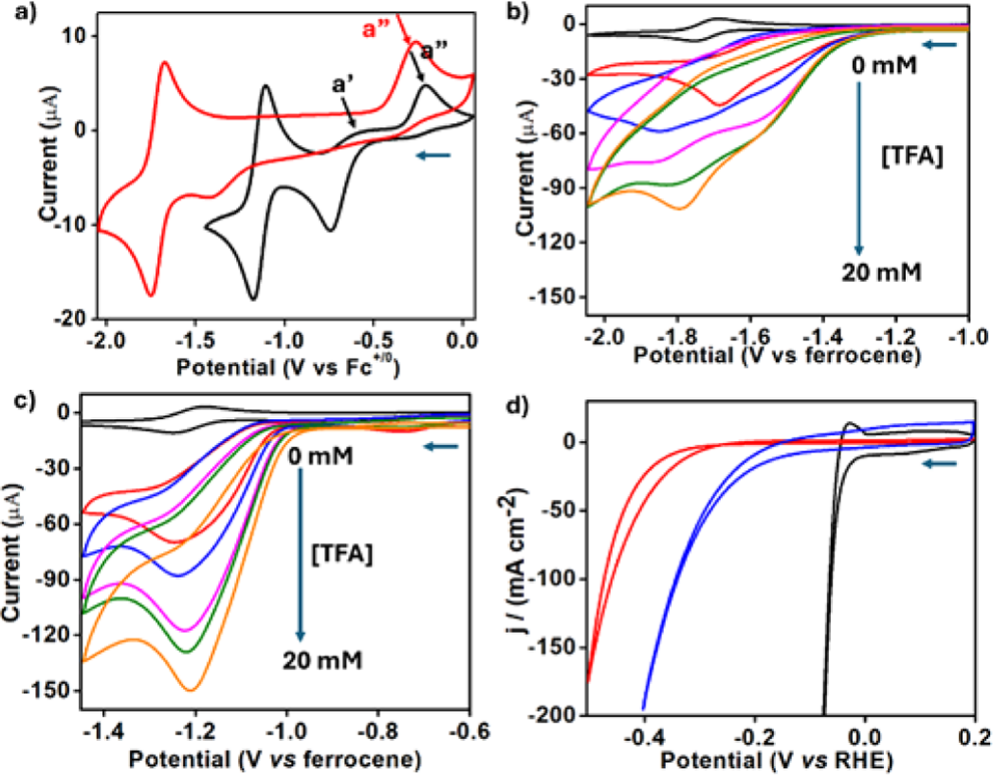
CV traces (0.5 mM substrate, 0.1 M TBAPF_6_/CH_3_CN/N_2_, 100 mV/s scan rate, Ag/AgCl reference electrode, potentials vs Fc/Fc^+^) of **1** (red) and **2** (black) with (a) no additives (a′ and a″ stand for reoxidation of the 6- and 4-coordinate cobalt­(II) complexes, respectively) and those of (b) **1** and (c) **2** in the presence of 0 mM (black), 4 mM (red) 8 mM (blue), 12 mM (pink), 16 mM (green), and 20 mM (orange) TFA. (d) CV traces (N_2_-saturated 0.5 M H_2_SO_4_ solution, scan rate 100 mV/s) obtained with cathodes composed of glassy carbon modified by BP2000 treated with either **1** (red) or **2** (blue) or by 20% Pt on Vulcan (black).

### HER Catalysis under Homogeneous Conditions

The activity of the complexes as HER catalysts was evaluated by recording cyclic voltammograms (CVs) in CH_3_CN with trifluoroacetic acid (p*K*_a_ of TFA in CH_3_CN = 12.7) as the proton source (Figure 3b and 3c).^43^ Control experiments performed without a catalyst confirmed that TFA does not induce any changes in current at potentials above −1.3 V (Figure S8). Under the same conditions, in the presence of **1**, the addition of 0–20 mM TFA led to quite ill-defined catalytic waves and only at rather negative potentials (Figure 3b). Much better results were obtained by employing **2** as the electrocatalyst (Figure 3c), as evidenced by (a) the shape of the catalytic waves; (b) the current; and (c) the onset potential of −0.96 V vs Fc/Fc^+^, which is very low relative to previous reports.^19,20,44,45^ The overpotential for HER catalysis by **2**, determined based on the potential at the middle of the catalytic wave obtained with 20 mM TFA, was 245 mV (the standard reduction potential of TFA is −0.89 V vs. ferrocene in acetonitrile, without taking into account the homoconjugation that may increase its effective acidity).^6,46^ The peak currents increased linearly with the concentrations of TFA, consistent with molecular catalysis, and a comparison of the catalytic activity of the complexes in terms of *i*_c_/*i*_p_ also identified **2** as the most potent catalyst (Figure S9). Complex **2** exhibited better HER activity than complex **1** in CH_3_CN using TFA as the proton source, with a turnover frequency (TOF) of 38.02 s^–1^ at 250 mV overpotential (Table S4 and S5). The evolution of hydrogen gas was confirmed and quantified by gas chromatography (Figure S10) after 30 min of constant potential electrolysis (CPE) at −1.2 V vs Fc/Fc^+^ in acetonitrile solution containing 0.5 mM catalyst and 100 mM TFA, with glassy carbon as the working electrode. Examination of the reaction mixtures by UV–vis spectroscopy and thin-layer chromatography before and after CPE (Figure S11), together with linear charge-time correlations observed for all catalysts, served to indicate that the complexes are efficient and durable HER catalysts.^47^ The charge that was passed throughout a 30 min electrolysis process with **2** as the catalyst was 175 mC (Figure S12), which may be translated to a Faradaic efficiency of 86%.

### Heterogeneous HER Catalysis of Acidic Water

Encouraged by the above-described results, **2** was studied for heterogeneous HER catalysis in an aqueous solution (Figure 3d) for applicative purposes. Metallocorroles immobilized on carbon were studied previously by our group, and **1** adsorbed on carbon was characterized by XPS.^19,28^ **2** was now found to be completely adsorbed on Black Pearls BP2000 (Figure S13), selected since it has a very large surface area (1537 m^2^/g) and pores of 20–25 nm, big enough to adsorb a few corroles.^48^ Heterogeneous HER catalysis was performed by modifying glassy carbon electrodes with inks containing 0.8 mg of catalyst adsorbed on 10 mg BP2000.^49^ The cobalt corroles were successfully adsorbed onto BP2000 carbon using 2-propanol (IPA) as the dispersion medium to obtain corrole-modified carbon materials. The detailed synthesis procedure is provided in Supporting Information. Scanning electron microscopy (SEM) images of the modified carbon materials, along with their corresponding EDS spectra, are shown in Figure S14. Compared to the unmodified BP2000, the corrole-modified samples display a slightly roughened surface morphology, which is attributed to the adsorption of cobalt corroles. Furthermore, elemental mapping via EDS (Figure S14) confirms the presence of Co and F for **1** and Co, F, and Br for **2**. These results provide strong evidence for the successful immobilization of cobalt corrole complexes on the BP2000 surface. Furthermore, the weight percentage of Co indicates that BP2000 contains about 8% of complex **1** and about 12% of complex **2**. These results are in good agreement with the UV–vis spectra measurements (Figure S13), which indicate that corroles **1** and **2** are totally adsorbed on BP2000 (0.8 mg/10 mg, 8%). Employment of a cathode that contained catalyst-free BP2000 revealed no electrocatalytic proton reduction of acidic water down to −0.6 V, while the activity in terms of catalytic onset potential and current varied very much as a function of the adsorbed molecular catalysts: low for **1** and much better for **2** (Figure 3d). The results obtained in the latter case, with 8% catalyst loading, were quite close to those observed with 20% platinum on Vulcan. The reaction kinetics of the prepared samples were evaluated through a Tafel slope analysis. As illustrated in Figure S15, the Tafel slope for **2** was found to be 106 mV dec^–1^, which is smaller than that for **1** (128 mV dec^–1^), and its exchange current density is 3.98 μA/cm^2^ (6.76 μA/cm^2^ for **1**). Clearly visible hydrogen bubbles formed on the surface of the **2**-modified cathode, immersed in a 0.5 M sulfuric acid solution under a −0.4 V applied potential vs RHE. Chronoamperometry and gas chromatography for product quantification uncovered a Faradaic efficiency (FE) of 97% and stability of greater than 10 h (Figure S16).

## Conclusions

Bromination of the cobalt­(III) complex of the already electron-poor third-generation corrole **1** induces very significant property changes: complex **2** is characterized by a very distorted macrocycle, coordinates pyridine axial ligands more strongly and in a perpendicular orientation, displays a highly red-shifted electronic spectrum, and is easier to reduce by 0.53–0.64 V. A beneficial outcome of the latter aspect is that electrocatalytic reduction of TFA in organic solvent in the presence of **2**, as well as of acidic water by a **2**-modified cathode, proceeds with large Faradaic efficiency and low overpotentials. Future studies will focus on the bromination of even more electron-poor cobalt­(III) corrole complexes (like those with CN rather than CF_3_ on the *meso*-C positions) to elucidate the role of changes in coordination chemistry on catalytic performance and possibly also on the reaction mechanism.

## Experimental Section

### Syntheis of 5,10,15-Tris­(trifluoromethyl)­corrolato Cobalt­(III) (bis)­Pyridine (1)

Complex **1** was synthesized according to the procedure previously reported.^34^

### Synthesis of Complex 2

A procedure was adapted from the literature as follows:^50^ **1** (70 mg, 0.097 mmol) was dissolved in CHCl_3_ (30 mL) in a round-bottom flask equipped with a magnetic stirrer bar. To the stirred reaction mixture at room temperature was added bromine (296 μL, 52.5 equiv) dissolved in CHCl_3_ (12 mL) with a dropping funnel over 30 min. After an additional 1 h of stirring, pyridine (561 mL, 63 equiv) dissolved in CHCl_3_ (12 mL) was added with a dropping funnel over 15 min. The reaction mixture was stirred at room temperature overnight, shaken with 20 mL of 20% w/v aqueous sodium metabisulfite, dried over MgSO_4_ and filtered. The solvent was evaporated, and the product was purified by silica gel column chromatography with hexane:ethyl acetate:pyridine (8:2:0.1) as the eluent. After recrystallization from *n*-hexane/CH_2_Cl_2_/pyridine, **2** was obtained in 52% yield (57.8 mg, 0.051 mmol). Elemental analysis calculated (found) for C_32_H_10_Br_8_CoF_9_N_6_ (**2**): C, 28.52 (28.63); H, 0.75 (0.68); N, 6.24 (6.30). ^1^H NMR (400 MHz, C_6_D_6_) δ 4.81 (t, *J* = 7.5 Hz, 1H), 3.79 (t, *J* = 7.0 Hz, 2H), 1.67 (d, *J* = 5.7 Hz, 2H). ^19^F NMR (377 MHz, CDCl_3_) δ −33.54 (b, 9F) ppm. HRMS (APCI, positive mode) for C_22_N_4_Br_8_F_9_Co: *m*/*z* 1189.2696 (calculated), 1189.2732 (observed). UV–vis (DCM): λ_max_ (ε) = 503 (54800), 621 (7083), 670 (12224).

## Supplementary Material



## Notes

NMR (^1^H and ^19^F) and HRMS spectra of all new compounds/complexes and additional electrochemical data (PDF)

## References

[1] Marinescu S. C., Winkler J. R., Gray H. B. (2012). Molecular Mechanisms of Cobalt-Catalyzed
Hydrogen Evolution. Proc. Natl. Acad. Sci. U.
S. A.

[2] Gray H. B. (2009). Powering
the Planet with Solar Fuel. Nat. Chem.

[3] Espitalier-Noël, M. ; Fonseca, J. ; Fraile, D. ; Muron, M. ; Pawelec, G. ; Santos, S. ; Staudenmayer, O. Clean Hydrogen Monotor 2024, https://hydrogeneurope.eu/wpcontent/uploads/2023/10/Clean_Hydrogen_Monitor_11-2023.

[4] Beyene B. B., Hung C. H. (2020). Recent Progress
on Metalloporphyrin-Based Hydrogen
Evolution Catalysis. Coord. Chem. Rev..

[5] Helm M. L., Stewart M. P., Bullock R. M., DuBois M. R., DuBois D. L. (2011). A Synthetic
Nickel Electrocatalyst with a Turnover Frequency above 100,000 s^–1^ for H_2_ Production. Science.

[6] Jacques P. A., Artero V., Pécautb J., Fontecave M. (2009). Cobalt and
Nickel Diimine-Dioxime Complexes as Molecular Electrocatalysts for
Hydrogen Evolution with Low Overvoltages. Proc.
Natl. Acad. Sci. U. S. A..

[7] Barrozo A., Orio M. (2019). Molecular Electrocatalysts for the Hydrogen Evolution Reaction: Input
from Quantum Chemistry. ChemSuschem.

[8] Moschkowitsch W., Lori O., Elbaz L. (2022). Recent Progress
and Viability of
PGM-Free Catalysts for Hydrogen Evolution Reaction and Hydrogen Oxidation
Reaction. ACS Catal..

[9] Lv Z. Y., Yang G., Ren B. P., Liu Z. Y., Zhang H., Si L. P., Liu H. Y., Chang C. K. (2023). Electrocatalytic
Hydrogen Evolution of the Cobalt Triaryl Corroles Bearing Hydroxyl
Groups. Eur. J. Inorg. Chem..

[10] Alvarez-Hernandez J. L., Sopchak A. E., Bren K. L. (2020). Buffer pKa Impacts
the Mechanism
of Hydrogen Evolution Catalyzed by a Cobalt Porphyrin-Peptide. Inorg. Chem..

[11] Li X., Lei H., Xie L., Wang N., Zhang W., Cao R. (2022). Metalloporphyrins
as Catalytic Models for Studying Hydrogen and Oxygen Evolution and
Oxygen Reduction Reactions. Acc. Chem. Res..

[12] Peng X., Zhang M., Qin H., Han J., Xu Y., Li W., Zhang X.-P., Wei Zhang W., Apfel U.-P., Cao R. (2024). Switching
Electrocatalytic Hydrogen Evolution Pathways through Electronic Tuning
of Copper Porphyrins. Angew. Chem., Int. Ed..

[13] Li X., Lv B., Zhang X.-P., Jin X., Guo K., Zhou D., Bian H., Zhang W., Apfel U.-P., Cao R. (2022). Introducing
Water-Network-Assisted Proton Transfer for Boosted Electrocatalytic
Hydrogen Evolution with Cobalt Corrole. Angew.
Chem., Int. Ed..

[14] Peng X., Han J., Li X., Liu G., Xu Y., Peng Y., Nie S., Li W., Li X., Chen Z., Peng H., Cao R., Fang Y. (2023). Electrocatalytic hydrogen evolution with a copper porphyrin
bearing *meso-*(o-carborane) substituents. Chem. Commun..

[15] Lin Q., Li L., Li M., Magwaza T., Mack J., Nyokong T., Zhu W., Liang X. (2025). Controlling the Electron Conductive Pathways of Co^III^-triarycorroles on the Gold Electrode Surface and their
Electrocatalyzed Hydrogen Evolution *Inorg*. Chemica. Acta.

[16] Gross Z., Gray H. (2006). How Do Corroles Stabilize High Valent Metals?. Comments Inorg. Chem..

[17] Goldberg D. P. (2007). Corrolazines:
New Frontiers in High-Valent Metalloporphyrinoid Stability and Reactivity. Acc. Chem. Res..

[18] Ramdhanie B., Telser J., Caneschi A., Zakharov L. N., Rheingold A. L., Goldberg D. P. (2004). An Example of O_2_ Binding in a Cobalt­(II)
Corrole System and High-Valent Cobalt-Cyano and Cobalt-Alkynyl Complexes. J. Am. Chem. Soc..

[19] Kumar A., Fite S., Raslin A., Kumar S., Mizrahi A., Mahammed A., Gross Z. (2023). Beneficial Effects on the Cobalt-Catalyzed
Hydrogen Evolution Reaction Induced by Corrole Chelation. ACS Catal..

[20] Chen Q. C., Fite S., Fridman N., Tumanskii B., Mahammed A., Gross Z. (2022). Hydrogen Evolution Catalyzed by Corrole-Chelated
Nickel Complexes, Characterized in All Catalysis-Relevant Oxidation
States. ACS Catal..

[21] Lei H., Fang H., Han Y., Lai W., Fu X., Cao R. (2015). Reactivity and Mechanism Studies
of Hydrogen Evolution Catalyzed
by Copper Corroles. ACS Catal..

[22] Sudhakar K., Mizrahi A., Kosa M., Fridman N., Tumanskii B., Saphier M., Gross Z. (2017). Effect of
Selective CF_3_ Substitution on the Physical and Chemical
Properties of Gold Corroles. Angew. Chem., Int.
Ed..

[23] Mondal B., Sengupta K., Rana A., Mahammed A., Botoshansky M., Dey S. G., Gross Z., Dey A. (2013). Cobalt Corrole Catalyst
for Efficient Hydrogen Evolution Reaction from H_2_O under
Ambient Conditions: Reactivity, Spectroscopy, and Density Functional
Theory Calculations. Inorg. Chem..

[24] Mahammed A., Mondal B., Rana A., Dey A., Gross Z. (2014). The Cobalt
Corrole Catalyzed Hydrogen Evolution Reaction: Surprising Electronic
Effects and Characterization of Key Reaction Intermediates. Chem. Commun..

[25] Bhyrappa P., Krishnan V. (1991). Octabromotetraphenylporphyrin and Its Metal Derivatives:
Electronic Structure and Electrochemical Properties. Inorg. Chem..

[26] Mahammed A., Tumanskii B., Gross Z. (2011). Effect of Bromination on the Electrochemistry, Frontier Orbitals,
and Spectroscopy of Metallocorroles. J. Porphyr.
Phthalocyanines.

[27] Chen Q., Soll M., Mizrahi A., Saltsman I., Fridman N., Saphier M., Gross Z. (2018). One-Pot Synthesis
of Contracted and
Expanded Porphyrins with Meso -CF_3_ Groups. Angew. Chem., Int. Ed..

[28] Raslin A., Douglin J. C., Kumar A., Fernandez-Dela-Mora M., Dekel D. R., Gross Z. (2023). Size and Electronic Effects on the
Performance of (Corrolato)­Cobalt-Modified Electrodes for Oxygen Reduction
Reaction Catalysis. Inorg. Chem..

[29] Kumar A., Yadav P., Majdoub M., Saltsman I., Fridman N., Kumar S., Kumar A., Mahammed A., Gross Z. (2021). Corroles:
The Hitherto Elusive Parent Macrocycle and Its Metal Complexes. Angew. Chem., Int. Ed..

[30] Sudhakar K., Mahammed A., Fridman N., Gross Z. (2017). Iodinated Cobalt Corroles. J. Porphyr. Phthalocyanines.

[31] Simkhovich L., Mahammed A., Goldberg I., Gross Z. (2001). Synthesis and Characterization
of Germanium, Tin, Phosphorus, Iron, and Rhodium Complexes of Tris­(Pentafluorophenyl)­Corrole,
and the Utilization of the Iron and Rhodium Corroles as Cyclopropanation
Catalysts. Chem. Eur. J..

[32] Ganguly S., Conradie J., Bendix J., Gagnon K. J., McCormick L. J., Ghosh A. (2017). Electronic Structure
of Cobalt-Corrole-Pyridine Complexes: Noninnocent
Five-Coordinate Co­(II) Corrole-Radical States. J. Phys. Chem. A.

[33] Simkhovich L., Galili N., Saltsman I., Goldberg I., Gross Z. (2000). Coordination
Chemistry of the Novel 5,10,15-Tris­(Pentafluorophenyl)­Corrole: Synthesis,
Spectroscopy, and Structural Characterization of Its Cobalt­(III),
Rhodium­(III), and Iron­(IV) Complexes. Inorg.
Chem..

[34] Yadav P., Khoury S., Fridman N., Sharma V. K., Kumar A., Majdoub M., Kumar A., Diskin-Posner Y., Mahammed A., Gross Z. (2021). Trifluoromethyl Hydrolysis
En Route
to Corroles with Increased Druglikeness. Angew.
Chem., Int. Ed..

[35] Sahoo D., Mazumdar R., Pramanik S., Banerjee S., Patra R., Rath S. P. (2023). Modulation of Iron Spin States in Highly Distorted
Iron­(III) Porphyrins: H-Bonding Interactions and Implications in Hemoproteins. Dalt. Trans.

[36] Bogdanovi G. A., Medakovi V., Mil M. K., Zari S. D. (2004). Intramolecular C–H
π Interactions in Metal-Porphyrin Complexes. Int. J. Mol. Sci..

[37] Safo M. K., Nesset M. J. M., Walker F. A., Debrunner P. G., Scheidt W. R. (1997). Models of the Cytochromes. Axial
Ligand Orientation
and Complex Stability in Iron­(II) Porphyrinates: The Case of the Noninteracting
dπ Orbitals. J. Am. Chem. Soc..

[38] Saini A., Sharma V. K., Fridman N., Mahammed A., Gross Z. (2024). Triscyanocorrole,
the Newest and Most Intriguing Member of the C1-Substituted Corrole
Family. Chem.–Eur. J..

[39] Chen H., Huang D. L., Hossain M. S., Luo G. T., Liu H. Y. (2019). Electrocatalytic
Activity of Cobalt Tris­(4-Nitrophenyl)­Corrole for Hydrogen Evolution
from Water. J. Coord. Chem..

[40] Lin H., Hossain M. S., Zhan S. Z., Liu H. Y., Si L. P. (2020). Electrocatalytic
Hydrogen Evolution Using Triaryl Corrole Cobalt Complex. Appl. Organom. Chemis..

[41] Liang Y. Y., Li M. Y., Shi L., Lin D. Z., Zhan S. Z., Liu H. Y. (2021). Electrocatalytic
Hydrogen Evolution by Cobalt Triaryl
Corroles with Appended Ester and Carboxyl on the 10-Phenyl Group. J. Coord. Chem..

[42] Osterloh W. R., Desbois N., Fleurat-Lessard P., Fang Y., Gros C. P., Kadish K. M. (2023). Altering the Site of Electron Abstraction in Cobalt
Corroles via Meso-Trifluoromethyl Substituents. Inorg. Chem..

[43] Felton G. A. N., Glass R. S., Lichtenberger D. L., Evans D. H. (2006). Iron-Only Hydrogenase
Mimics. Thermodynamic Aspects of the Use of Electrochemistry to Evaluate
Catalytic Efficiency for Hydrogen Generation. Inorg. Chem..

[44] Li X., Lei H., Guo X., Zhao X., Ding S., Gao X., Zhang W., Cao R. (2017). Graphene-Supported Pyrene-Modified
Cobalt Corrole with Axial Triphenylphosphine for Enhanced Hydrogen
Evolution in pH 0–14 Aqueous Solutions. ChemSuschem.

[45] Rana A., Mondal B., Sen P., Dey S., Dey A. (2017). Activating
Fe­(I) Porphyrins for the Hydrogen Evolution Reaction Using Second-Sphere
Proton Transfer Residues. Inorg. Chem..

[46] Fourmond V., Jacques P. A., Fontecave M., Artero V. (2010). H_2_ Evolution
and Molecular Electrocatalysts: Determination of Overpotentials and
Effect of Homoconjugation. Inorg. Chem..

[47] Mahammed A., Giladi I., Goldberg I., Gross Z. (2001). Synthesis and Structural
Characterization of a Novel Covalently-Bound Corrole Dimer. Chem. Eur. J..

[48] Levy N., Lori O., Gonen S., Mizrahi M., Ruthstein S., Elbaz L. (2020). The Relationship of
Morphology and Catalytic Activity: A Case Study
of Iron Corrole Incorporated in High Surface Area Carbon Supports. Carbon.

[49] Shahaf Y., Mahammed A., Raslin A., Kumar A., Farber E. M., Gross Z., Eisenberg D. (2022). Orthogonal
Design of Fe–N4
Active Sites and Hierarchical Porosity in Hydrazine Oxidation Electrocatalysts. ChemElectrochem.

[50] Norheim H. K., Schneider C., Gagnon K. J., Ghosh A. (2017). One-Pot Synthesis of
a Bis-Pocket Corrole through a 14-Fold Bromination Reaction. Chem. Open.

