# Supplementary material for: Peripheral Bromination for Strongly Affecting the Structural, Electronic, and Catalytic Properties of Cobalt Corroles
Source: Inorg Chem. 2025 May 28;64(22):11069–74. doi: 10.1021/acs.inorgchem.5c01310 (PMC12152934; doi:10.1021/acs.inorgchem.5c01310)
Supplement: Supplementary file 1 [file ic5c01310_si_001.pdf]

Supporting information

Peripheral Bromination for Strongly Affecting the Structural, Electronic, and Catalytic Properties of Cobalt Corroles

Sachin Kumar,[1] Arik Raslin,[1] Sruti Mondal,[1] Amir Mizrahi,[1,2] Natalia Fridman,[1] Atif Mahammed,[1] and Zeev Gross[1]*

[1]Schulich Faculty of Chemistry, Technion–Israel Institute of Technology, Haifa 32000 Israel

[2] Department of Chemistry, Nuclear Research Centre-Negev, Beer Sheva 9001 Israel

*****Corresponding Author**. Email: chr10zg@technion.ac.il**

**Table of Contents**	**Page**
Chemicals and Instrumentation	S3
**Figure S1**: ^{1}H NMR spectrum (400 MHz, CD_3CN) of **1**.	S8
**Figure S2**: ^{19}F NMR spectrum (377 MHz, CD_3CN) of **1**.	S8
**Figure S3**: ^{1}H NMR spectrum (400 MHz, C_6D_6) of **2**.	S9
**Figure S4**: ^{19}F NMR spectrum (377 MHz, C_6D_6) of **2**.	S9
**Figure S5:** HRMS of **2**, APCI positive mode.	S10
**Figure S6:** CVs of a) **1**, b) **2**, in CH_3CN as a solvent that contains 0.1M $TBAPF_6$ and at different scan rates of 100 (black), 200 (red), 300 (blue), 400 (pink), 500 (green), and 600 (dark blue) mV/sec. The plot of peak current *vs.* square root of scan rate 2nd reduction (black), oxidation (red) using Randles-Ševčik equation: c) **1** and d) **2.**	S10
**Figure S7:** Spectroelectrochemical measurements of 0.125 mM **2** during its reduction at an applied potential of a) -0.6 V vs. Ag/AgCl and reoxidation (b) at applied potential of 0.6 V vs. Ag/AgCl (c) reduction at applied potential of -1.1 V vs. Ag/AgCl (d) reoxidation at applied potential of -0.4 V vs. Ag/AgCl in acetonitrile containing 0.2M TBAP as electrolyte.	S11
**Figure S8:** Cyclic voltammograms of 0-20 mM TFA without catalyst. These cyclic voltammograms were recorded in CH_3CN solution containing 0.1 M $TBAPF_6$, on a glassy carbon electrode as a working electrode at scan rate of 100 mVs^{-1}. Potentials are listed versus Fc/Fc^+.	S12
**Figure S9:** The dependence of i_c/i_p on the concentration of TFA in reactions catalyzed by **1** (black), **2** (red).	S12
**Figure S10:** GC-TCD read out of **2**.	S13
**Figure S11:** UV-Visible spectra of Cobalt(III) corroles a) **1** and b) **2**, before catalysis (black), before catalysis + TFA (red), and after catalysis (blue).	S13
**Figure S12:** Plot of charge *vs.* time of no catalyst only 100 mM TFA (blue) with 0.5 mM of **1** (red) and **2** (black) during 1800 seconds of electrolysis at an applied potential of −1.2 V *vs.* Fc/Fc^+.	S14
**Figure S13:** Electronic spectra before (black traces) and after (red traces) adsorption of 0.8 mg of a) **1** and b) **2** on 10 mg of BP2000 from 1 mL solution of isopropanol.	S14
**Figure S14:** SEM images of (a) bare BP2000, (c) 1Co@ BP2000, and (e) 2Co@ BP2000. EDAX patterns of (b) bare BP2000, (d) 1Co@ BP2000, and (f) 2Co@ BP2000.	S15
**Figure S15:** Tafel plots corresponding to 1Co@BP2000 (Red) and 2Co@BP2000 (Blue).	S16
**Figure S16:** The bulk electrolysis obtained with cathodes composed of glassy carbon modified by BP2000 **2** at an applied potential of -0.6 V (in N_2-saturated 0.5 M H_2SO_4 solution, scan rate 100 mV/s), a) the electric current *vs.* time for 10 hours and (b) electric charge *vs.* time.	S17

Experimental details

Chemicals and Instrumentation

All common chemical reagents and solvents were bought from commercial sources and purified before use according to established protocols. Dichloromethane A.R, n-Hexanes A.R, diethyl ether extra dry, and HPLC grade acetonitrile were purchased from J. T. Baker. Pyrrole (99%, extra pure), halothane (≥ 99%), sodium dithionite (≥82%), sodium bicarbonate (for analysis), PIFA (97%), $Co(OAc)_2 \cdot 4H_2O$ (for metallation), and tetrabutylammonium hexafluorophosphate (≥ 99%) were purchased from Sigma. BP2000, and 20% Pt/Vulcan were purchased from the Fuel Cell Store company. The silica gel used for column chromatography was Kiesel gel 60, 230–400 mesh. Absorption spectra of synthesized corroles were recorded on Agilent Technologies Cary 8454 UV-vis spectrophotometer. Quartz cuvettes of 1.0 cm thickness were used to measure the samples. ^{1}H and ^{19}F NMR spectra were recorded on a Bruker Avance III 400 MHz spectrometer equipped with a 5 mm diameter of the sample and matching with a broad-band probe (BBFO) by z-gradients. The 400 NMR operates at a frequency of 400.4 MHz for proton (^{1}H) nuclei and 377 MHz for fluorine (^{19}F) nuclei. Chemical shifts are reported in ppm. C_6D_6 (δ = 7.1) and $CDCl_3$ (δ = 7.26) were applied as an internal standard. Mestrenova v6.0.2-5475 software was used for the analysis of NMR spectra. High-resolution mass spectra for the compounds were performed on a Bruker MaXis Impact mass spectrometer, using APCI (atmospheric pressure chemical ionization) direct probe in either positive or negative mode. After electrolysis, H_2 gas was extracted from headspace and measured by using a Sion GC-TCD detector with an HP-mole sieve column and N_2 as a carrier gas. A calibration curve between the area of the peak and the known volume of H_2 gas was used to determine the amount of H_2 produced by electrocatalysis of acidic solution of Co(III) corroles. The stationary homogeneous and heterogeneous experiments were performed on a PALMSENS EmStat3+ potentiostat. The out-of-plane displacements for the macrocyclic rings, **1** and **2** were analyzed using PorphyStruct. based on single-crystal XRD structures.

Crystallographic details

The single-crystal of dark green block material **2** from CH_2Cl_2/C_6H_6/pyridine (1:3:0.001) immersed in paratone–N oil and mounted on a Bruker Apex2 at 140K Data collection was performed using monochromated Mo Kα radiation λ = 0.71073 Å, using φ and ω scans to cover the Ewald sphere. Accurate cell parameters were obtained with the amount of indicated reflections. Using Olex2,[1] the structure was solved with the olex2.solve[2] structure solution

program using Charge Flipping and refined with the ShelXL[3] refinement package using Least Squares minimization. All non-hydrogen atoms were refined with anisotropic displacement parameters. The hydrogen atoms were refined isotropically on calculated positions using a riding model with their U_{iso} values constrained to 1.5 times the U_{eq} of their pivot atoms for terminal sp^3 carbon atoms and 1.2 times for all other carbon atoms. Software used for molecular graphics: Mercury 2022.3.0.

Table S1: Crystallographic data for 2.

**Crystal data**	**2**
CCDC number	2395470
Empirical formula	$C_{32}H_{10}Br_8CoF_9N_6$
Formula weight (gr/mol)	1347.67
Temperature (K)	140
Wavelength (Å)	0.71073
Crystal system	monoclinic
Space group	$P2_1/c$
a (Å)	18.5129(14)
b (Å)	14.7046(11)
c (Å)	41.849(3)
α (°)	90
β (°)	100.906(2)
γ (°)	90
Volume ($Å^3$)	11186.6(15)
Z	12
Calculated density (g/cm^3)	2.401
Absorption coefficient (mm^{-1})	9.114
F (000)	7584.0
Crystal size (mm^3)	0.21×0.18×0.18
2Θ range (°)	2.696-50.28
Reflections collected	82113
R_{int}	0.1066
Data/restraints/ parameters	19508/1719/1507
Goodness-of-fit on F^2	0.991
R_1, wR_2 [I> 2σ (I)]	0.0603, 0.1058
R_1. wR_2 [all data]	0.1470, 0.1299
Largest diff. peak/ hole/ e $Å^{-3}$	2.30/-1.30
Diffractometer	APEX2 (Bruker AXS)

Cyclic Voltammograms were performed in acetonitrile solutions of 0.5 mM complex and 0.1 M tetrabutylammonium hexafluorophosphate ($TBAPF_6$), equipped with a glassy carbon working electrode, and Ag/AgCl and Pt wire as reference and counter electrodes, respectively. The solutions were purged with nitrogen before all experiments.

Determination of diffusion coefficient (D_0) of Cobalt(III) corroles 1,2.[4]

From the Randles-Ševčík equation (eq. 1), for one electron diffusional process, it was possible to obtain an apparent diffusion coefficient, D_0.

$$i_p = 2.69 \times 10^5 A[cat]D_0^{\ 0.5}\ \upsilon^{\ 0.5} \qquad \text{(eq. 1)}$$

Here, i_p is the peak current (A), n is the total number of electrons transferred, A is the electrode area (cm^2), [cat] is the bulk concentration of the analyte (mole cm^{-3}), D_0 is the diffusion coefficient ($cm^2\ s^{-1}$), and υ is the scan rate ($V\ s^{-1}$).

From i_p vs. square root of scan rate plot, eq (1) can remodified as

Slope $=2.69 \times 10^5 A[cat]D_0^{\ 0.5}$

$D_0 = (slope/\ 2{\cdot}69{\times}10^5 A[cat])^{\ 2}$

1: [cat] = 0.0000005 moles/cm^3, Slope (Fig S6c) = 13.93×10^{-6}, $D_0 = 2.2 \times 10^{-6}$ cm^2/s in CH_3CN

2: [cat] = 0.0000005 moles/cm^3, Slope (Fig S6d) = 21.48×10^{-6}, $D_0 = 5.2 \times 10^{-6}$ cm^2/s in CH_3CN

Calculation of Faradaic Efficiency (F.E.) and Turnover number

For bulk electrolysis, cobalt(III) corroles (**1,2**) were used at -1.2 V vs. Fc^+/Fc at 50 Mm TFA solvent using 0.1 M nBu_4NPF_6 as a supporting electrolyte. Glassy carbon used as a working electrode, Pt wire used as a counter electrode, and Ag/AgCl was chosen as a reference electrode. Faradaic Efficiency (F. E.) was calculated by using the equation:

Faradaic Efficiency = (Moles of H_2 Quantified / Moles of H_2 Theoretical) × 100%

Faradaic Efficiency (F.E.) of **2** = (0.0007797)/ (0.0009067) × 100%

F. E. of **2** = 86%

Turnover number

The turnover number (TON) has been calculated using the equation shown below: [5]

TON = moles of hydrogen gas generated/moles of catalyst used

TON of **2** = 0.000779/0.000005 = 155.4 (~ 156)

Table S2. Redox events of cobalt(III) corroles **1** and **2**.

Cobalt(III) corroles	Irreversible reduction (V vs Fc/Fc^+)	Irreversible oxidation (V vs Fc/Fc^+)	Reversible reduction (V vs Fc/Fc^+)
**1**	-1.38	-0.2	-1.68
**2**	-0.74	-0.3	-1.14

Adsorption of the complexes on BP2000, ink preparation and its adsorption on the working electrode: 0.8 mg of metallocorrole was dissolved in 1 mL of isopropanol (IPA) by five-minute sonication. 10 mg BP2000 was added, and the solution was sonicated for 15 minutes and left for 24 hours stirring. After that, the catalyst was centrifuged for thirty minutes, the solution was separated, and the carbon support was dried at 45 °C in an oven overnight. Then, 1 mL of IPA was added, and the mixture was centrifuged again. The solution was removed and combined with the previous day's solution. The catalyst was dried overnight again at the same temperature. The ink consisted of 1 mg catalyst, 0.2 mL IPA, 0.8 mL doubly ionized (DI) water and 5 µL Nafion. The ink was sonicated for 30 minutes. 2.5 µL of the ink was drop casted on the working electrode, dried under air for 30 minutes and then at 45 °C in the oven for 30 minutes.

Table S3: X-ray structural parameters and data relevant for appreciating deviation from planarity.

		**Torsion angles (°)**						**Ref.**
		**Saddle**				**Ruffle py-Co-py**		
		**α**	**β**	**γ**	**δ**	**ψ**	**χ**	
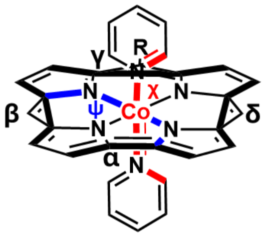	1: R = CF_3, X = H	2.14	7.93	-2.80	-9.43	6.81	0.7	6
	2: R = CF_3, X = Br	-4.68	-0.28	14.8	0.61	12.12	97.7	This work
	3: R = C_6F_5, X = H	-2.04	-7.24	5.24	12.44	-1.53	7.1	7
	4: R = C_6F_5, X = Br	-7.95	4.77	-1.05	13.21	8.19	84.3	8
	5: R = p-MeC_6H_4 X = H	0.61	5.89	-5.73	1.91	5.11	90.3	9

	6: R = p-MeC_6H_4 X = Br	9.30	-8.58	3.21	5.00	13.95	75.2	[9]

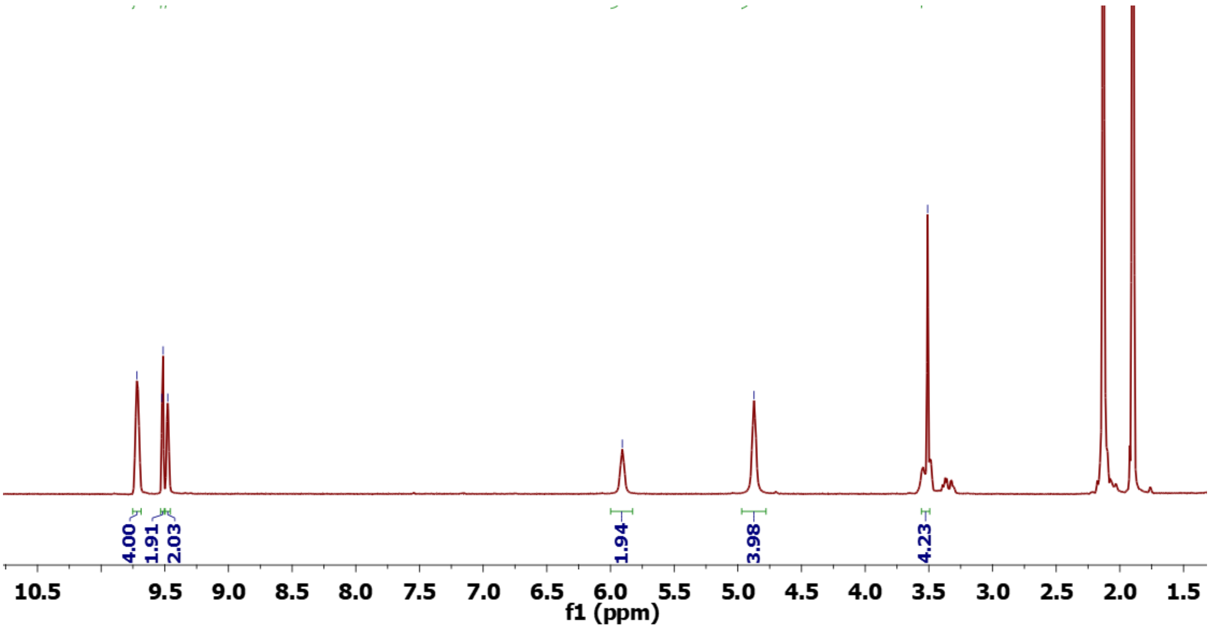

Figure S1: ^{1}H NMR spectrum (400 MHz, CD_3CN) of **1**.

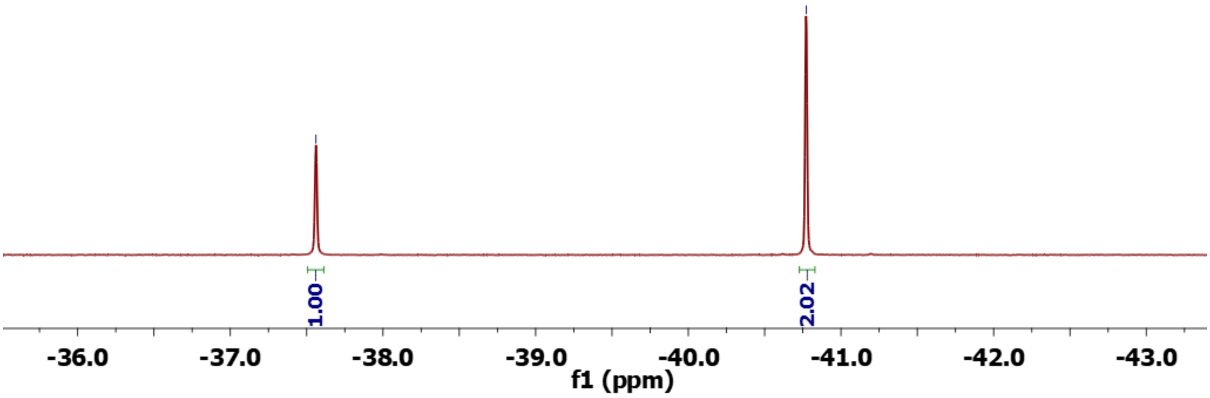

Figure S2: ^{19}F NMR spectrum (377 MHz, CD_3CN) of **1**.

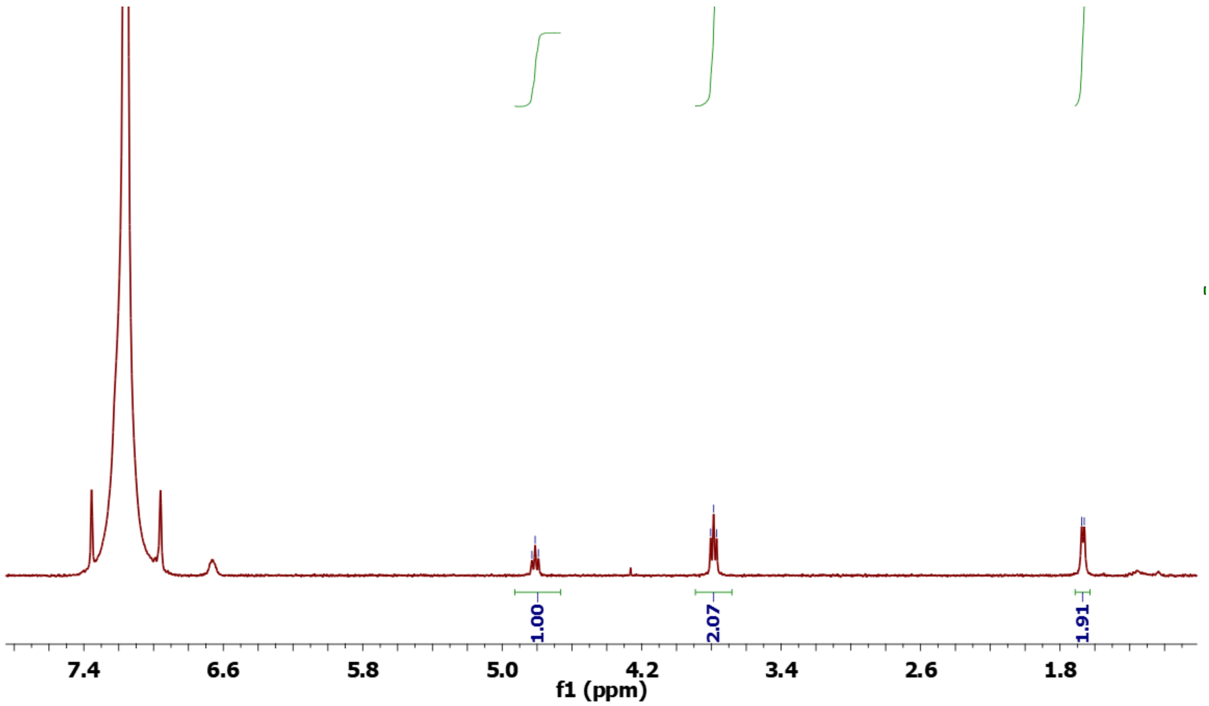

Figure S3: ^{1}H NMR spectrum (400 MHz, C_6D_6) of **2**.

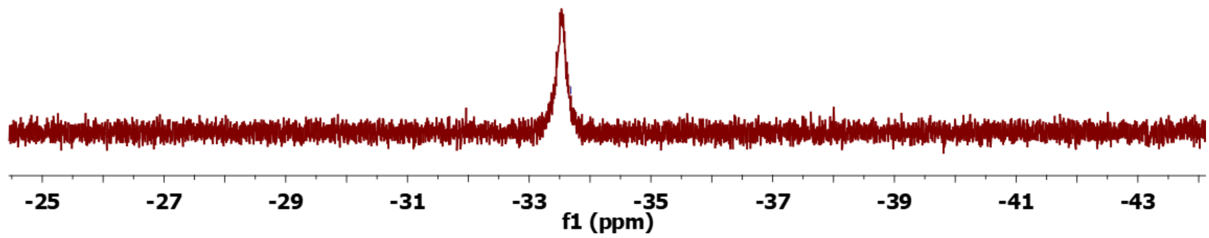

Figure S4: ^{19}F NMR spectrum (377 MHz, C_6D_6) of **2**.

Analysis Info

Analysis Name	D:\Data\Gross\Gr_6856n000002.d	Acquisition Date	13/01/2025 13:34:32
Method	APCI_pos_SolidProbe.m	Operator	Larisa Panz
Sample Name	CoTFCBr8Py2	Instrument	maXis impact 282001.00128
Comment			

Acquisition Parameter

Source Type	APCI	Ion Polarity	Negative	Set Nebulizer	1.2 Bar
Focus	Active	Set Capillary	4000 V	Set Dry Heater	120 °C
Scan Begin	50 m/z	Set End Plate Offset	-500 V	Set Dry Gas	1.5 l/min
Scan End	2000 m/z	Set Charging Voltage	2000 V	Set Divert Valve	Source
		Set Corona	2000 nA	Set APCI Heater	300 °C

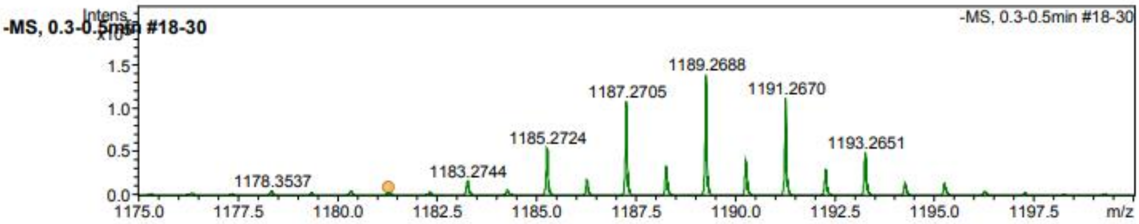

Meas. m/z	#	Ion Formula	m/z	err [ppm]	mSigma	# mSigma	Score	rdb	e⁻ Conf	N-Rule	|err| [mDa]
1181.2791	1	C14Br8CoF9N4O6	1181.2473	-26.9	52.0	5	0.00	8.5	even	-	31.8
	2	C12H8Br8CoF9N4O7	1181.3048	21.8	60.0	6	0.00	2.5	even	-	25.7
	3	C11H4Br8CoF9N4O8	1181.2684	-9.0	64.2	7	0.00	3.5	even	-	10.7
	4	C7H4Br8CoF9N4O11	1181.2532	-22.0	81.2	8	0.00	-0.5	even	-	25.9
	1	C22Br8CoF9N4	1181.2778	-1.1	22.1	1	100.00	16.5	even	-	1.3

Figure S5: HRMS of **2**, APCI positive mode.

Table S4. Experimental results of catalytic proton reduction by Co(III) corroles in homogeneous acetonitrile solution containing 20 mM TFA.

Complex	I_p(μA)	I_c(μA)	$(I_c/I_p)^2$	TOF (s^{-1})[a]	Overpotential (mV)[b]
**1**	-9.21	-101.34	121	23.47	790
**2**	-10.72	-150.40	196	38.02	250

- Potentials are in V *vs.* Fc/Fc^+ at scan rate of 100 mV/s (Working electrode: Glassy Carbon; Counter electrode: Pt wire). Catalyst concentration = 0.5 mM in acetonitrile containing 0.1 M TBAP electrolyte and 20 mM of TFA.
- [a] TOF = $k_{obs} = 1.94.\nu.(i_{cat}/i_p)^2$
- [b] Overpotential = $E_{cat} - [E^0\ (H^+/H_2)$ TFA]; [$E_0\ (H^+/H_2)$ TFA] = -0.89 V vs. $Fc^{+/0}$ in CH_3CN[10], E_{cat} is the potential in which the TOF was calculated.

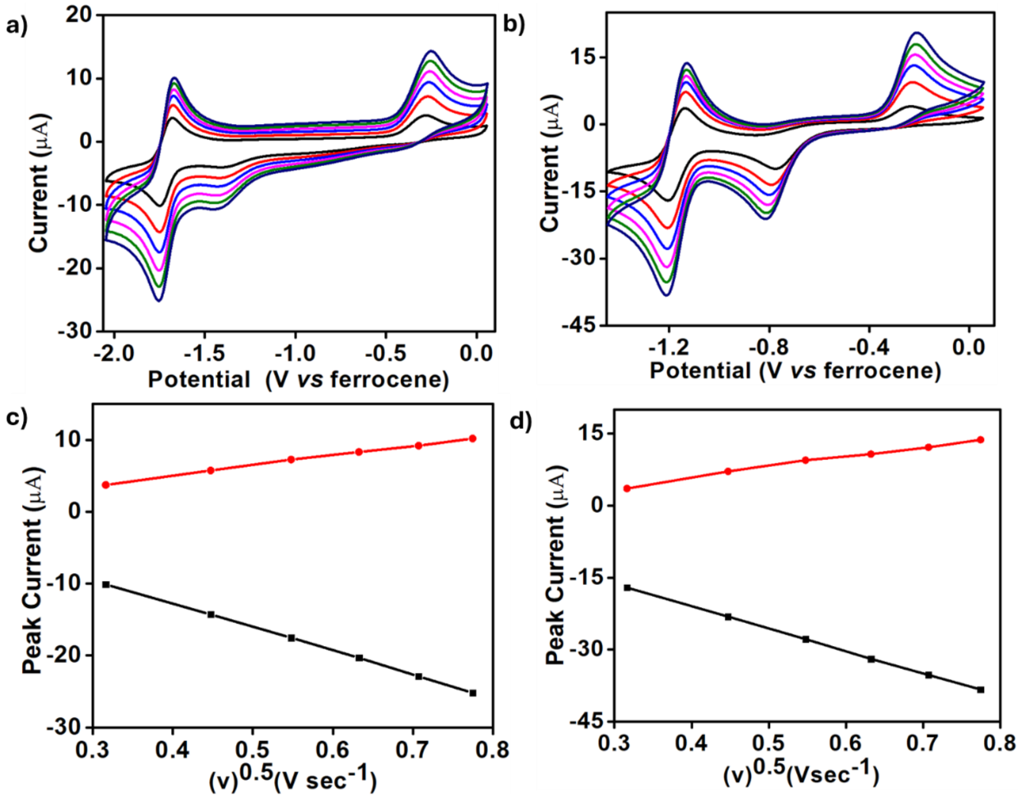

Figure S6: CVs of a) **1**, b) **2**, in CH_3CN as solvent that containing 0.1M $TBAPF_6$ and at different scan rates of 100 (black), 200 (red), 300 (blue), 400 (pink), 500 (green), and 600 (dark blue) mV/sec. Plot of peak current *vs.* square root of scan rate 2nd reduction (black), oxidation (red) using Randles-Ševčik equation: c) **1** and d) **2.**

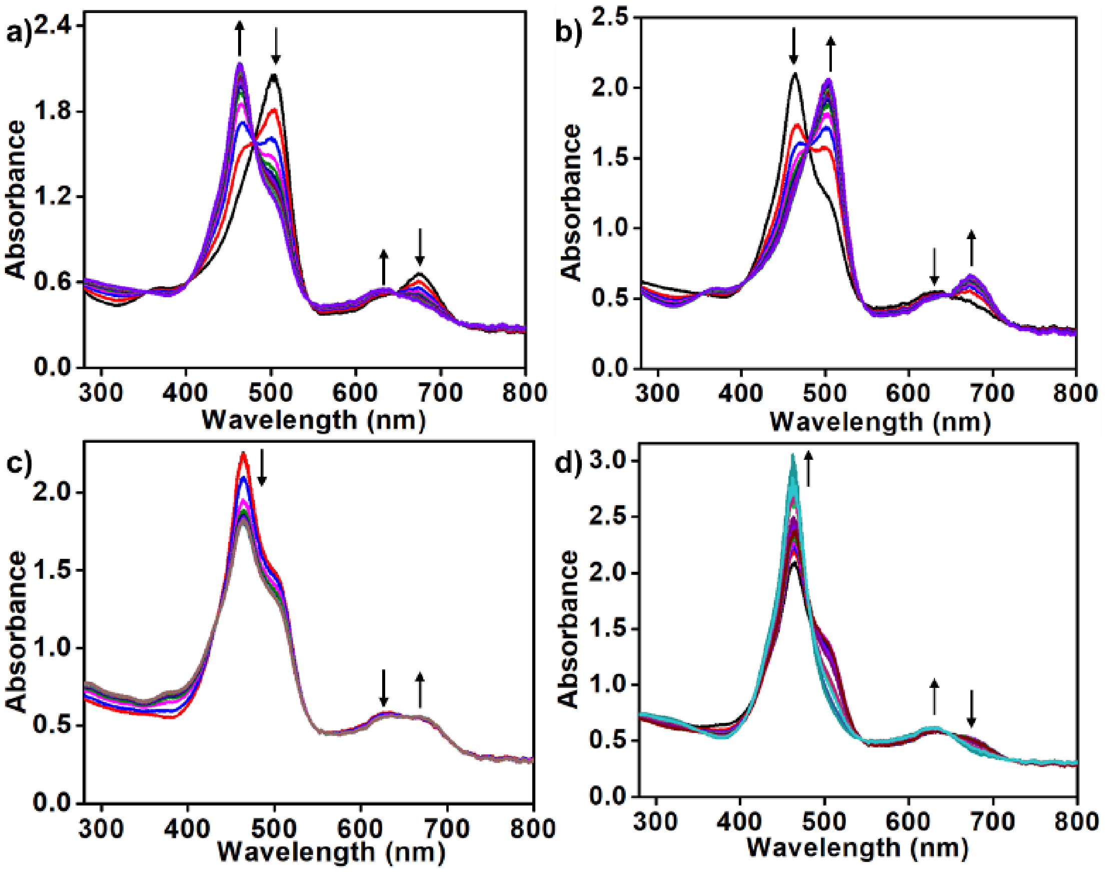

Figure S7: Spectroelectrochemical measurements of 0.125 mM **2** during its reduction at an applied potential of a) -0.6 V vs. Ag/AgCl and reoxidation (b) at applied potential of 0.6 V vs. Ag/AgCl (c) reduction at applied potential of -1.1 V vs. Ag/AgCl (d) reoxidation at applied potential of -0.4 V vs. Ag/AgCl in acetonitrile containing 0.2M TBAP as electrolyte.

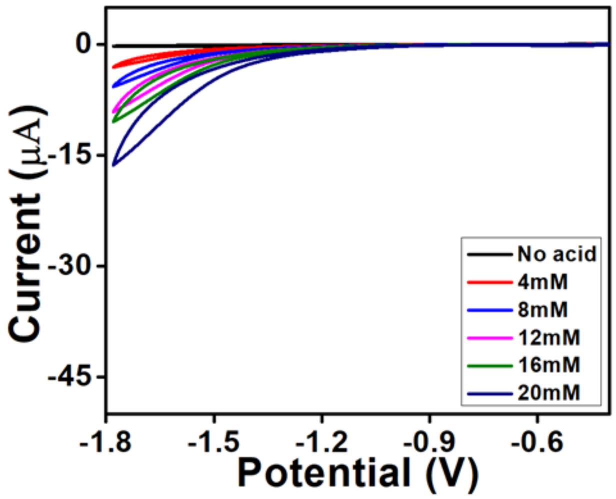

Figure S8: Cyclic voltammograms of 0-20 mM TFA without catalyst. These cyclic voltammograms were recorded in CH_3CN solution containing 0.1 M $TBAPF_6$, on a glassy carbon electrode as working electrode at scan rate of 100 mVs^{-1}. Potentials are listed versus Fc/Fc^+.

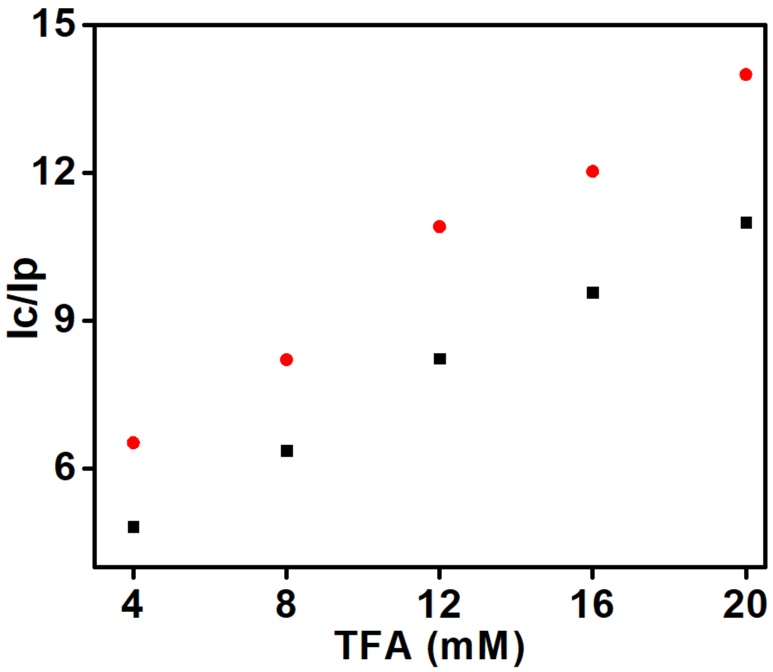

Figure S9: The dependence of i_c/i_p on the concentration of TFA in reactions catalyzed by **1** (black), **2** (red).

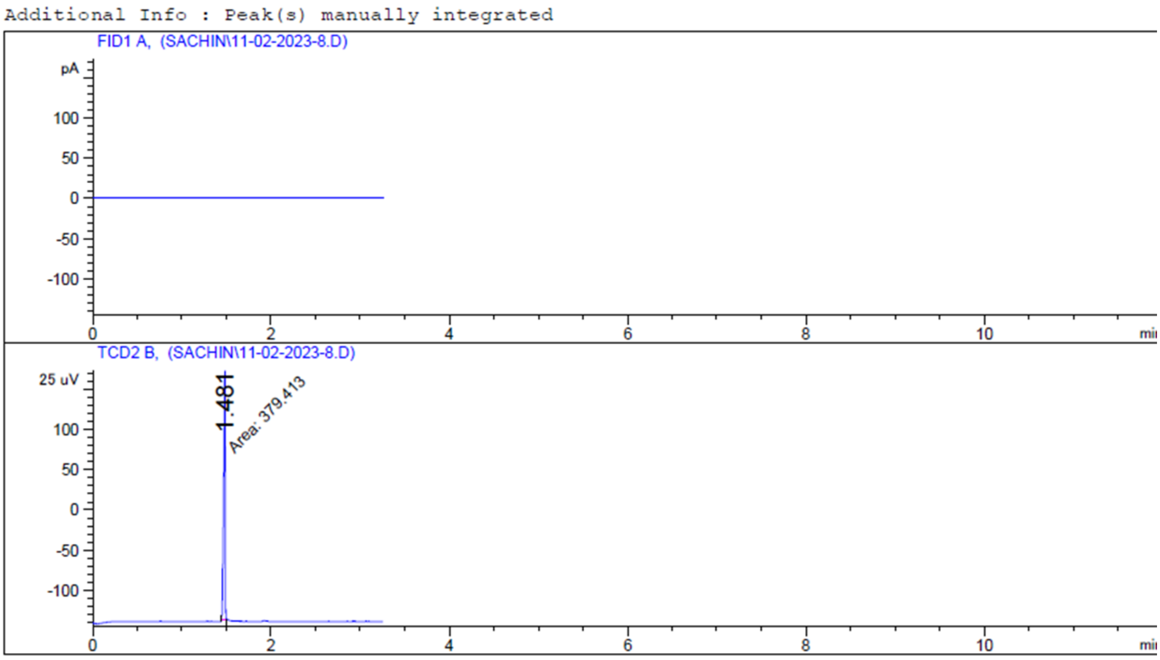

Peak #	RetTime [min]	Type	Width [min]	Area [25 uV*s]	Height [25 uV]	Area %
1	1.481	MM	0.0206	379.41257	306.96707	1.000e2

Totals : 379.41257 306.96707

Figure S10: GC-TCD read out of **2**.

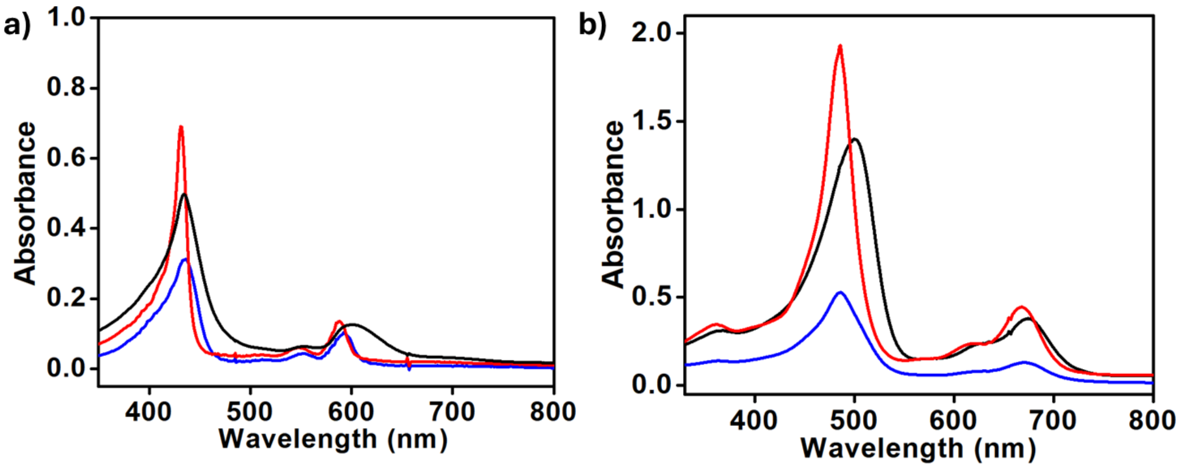

Figure S11: UV-Visible spectra of Cobalt(III) corroles a) **1** and b) **2**, before catalysis (black), before catalysis + TFA (red), and after catalysis (blue).

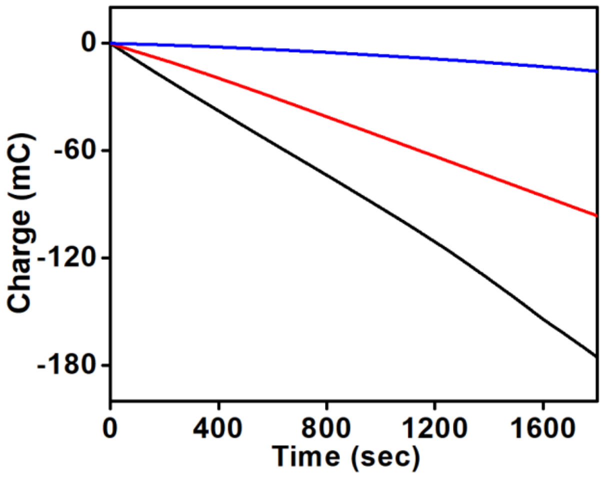

Figure S12: Plot of charge *vs.* time of no catalyst only 100 mM TFA (blue) with 0.5 mM of **1** (red) and **2** (black) during 1800 seconds of electrolysis at an applied potential of −1.2 V *vs.* Fc/Fc^{+}.

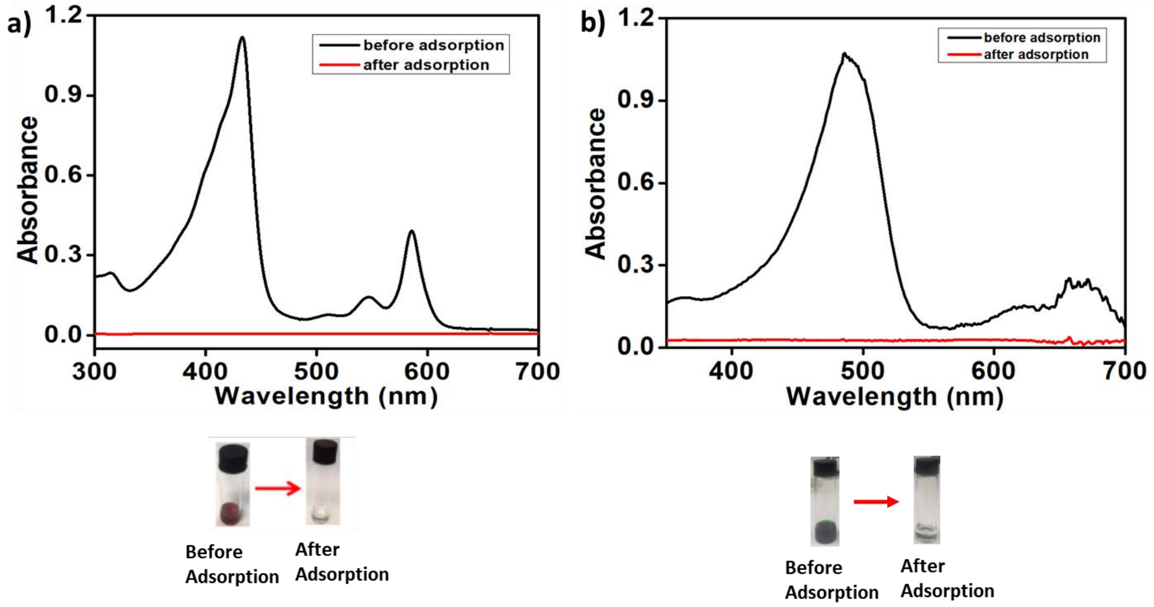

Figure S13: Electronic spectra before (black traces) and after (red traces) adsorption of 0.8 mg of a) **1** and b) **2** on 10 mg of BP2000 from 1 mL solution of isopropanol.

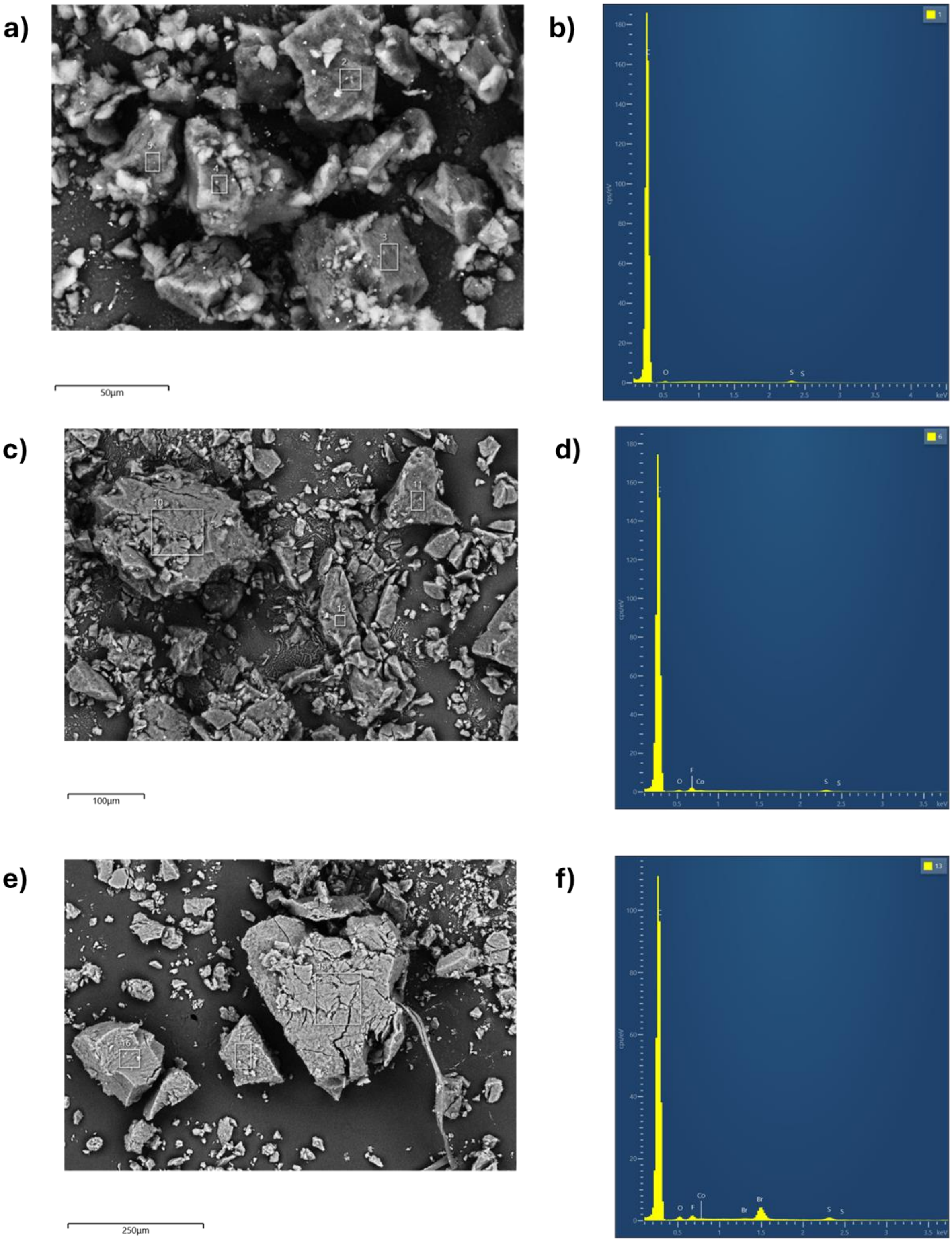

Figure S14: SEM images of (a) bare BP2000, (c) 1Co@ BP2000, and (e) 2Co@ BP2000. EDAX patterns of (b) bare BP2000, (d) 1Co@ BP2000, and (f) 2Co@ BP2000.

	Element	Weight%	Mole ratio
BP2000	C	98.31	
	O	1.07	
	S	0.91	
1@BP2000	Co	0.84	1
	F	1.72	6
2@BP2000	Co	0.61	1
	F	1.36	7
	Br	6.67	8

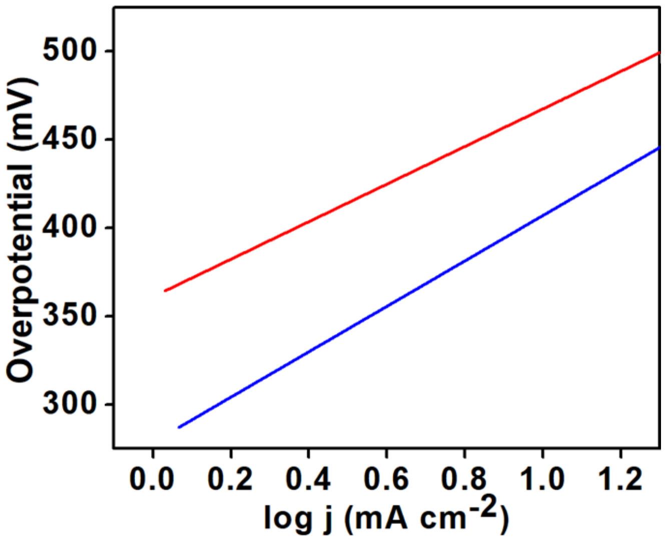

Figure S15: Tafel plots corresponding to 1Co@BP2000 (Red) and 2Co@BP2000 (Blue).

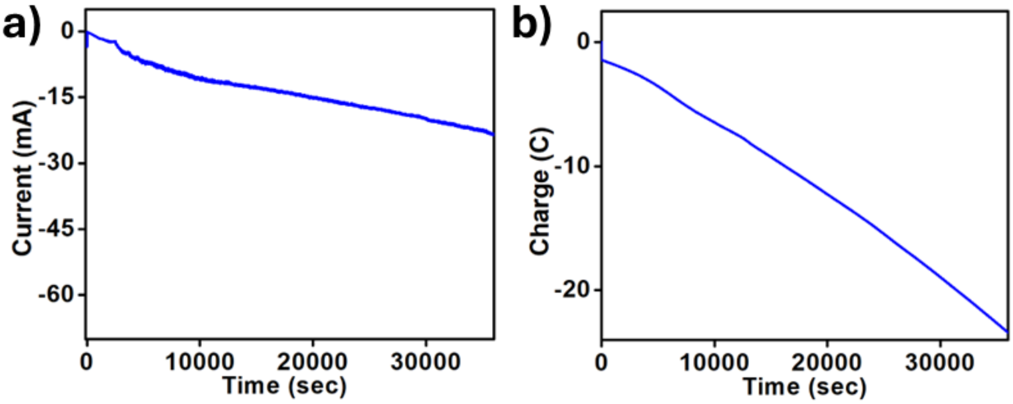

Figure S16: The bulk electrolysis obtained with cathodes composed of glassy carbon modified by BP2000 **2** at an applied potential of -0.6 V (in N_2-saturated 0.5 M H_2SO_4 solution, scan rate 100 mV/s), a) the electric current *vs.* time for 10 hours and (b) electric charge *vs.* time.

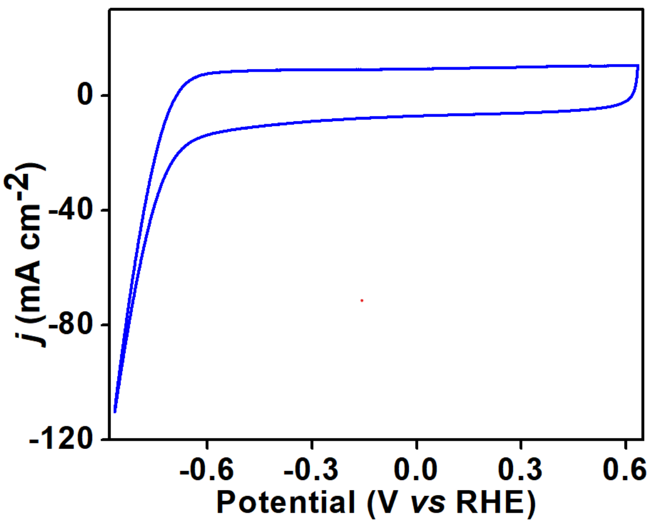

Figure S17: CV traces (N_2-saturated in phosphate buffer solution at pH 7.4, scan rate 100 mV/s) obtained with cathodes composed of glassy carbon modified by BP2000.

Table S5: TOF comparison for different metallocorrole complexes for electrocatalytic HER

Complex	TOF	Overpotential (mV)	Solution	Reference
**1**	23.47 s^{-1}	790	TFA in CH_3CN	This work
**2**	38.02 s^{-1}	250	TFA in CH_3CN	This work
(TPFC)$P^V(OH)_2$	31.75 s^{-1}	900	TFA in DMF	11
Fe(TPFC)Cl	274.2 h^{-1}	838	Water	12
2-PBHC-Co	1447.39 h^{-1}	1188	Neutral buffer media	13
3-PBHC-Co	1300.2 h^{-1}	1188	Neutral buffer media	13
4-PBHC-Co	1369.54 h^{-1}	1188	Neutral buffer media	13
Mn(TPFC)	1555.27 h^{-1}	838	Neutral buffer media	14
Ga(PFEC)	1193 h^{-1}	838	Neutral buffer media	15
Co(PFEC)	2533 h^{-1}	838	Neutral buffer media	15
Fe(PFEC)	1796 h^{-1}	838	Neutral buffer media	15
Co(TNPC)	188 h^{-1}	838	Neutral buffer media	16
CoF_8(TPFC)	600 s^{-1}	441	H_2SO_4	17

References:

(1) Dolomanov, O. V; Bourhis, L. J.; Gildea, R. J.; Howard, J. A. K.; Puschmann, H. OLEX2: A Complete Structure Solution, Refinement and Analysis Program. *J. Appl. Crystallogr.* **2009**, *42*, 339–341.

(2) Bourhis, L. J.; Dolomanov, O. V; Gildea, R. J.; Howard, J. A. K.; Puschmann, H. The Anatomy of a Comprehensive Constrained, Restrained Refinement Program for the Modern Computing Environment–Olex2 Dissected. *Acta Crystallogr. Sect. A Found. Adv.* **2015**, *71*, 59–75.

(3) Sheldrick, G. M. Crystal Structure Refinement with SHELXL. *Acta Crystallogr. Sect. C Struct. Chem.* **2015**, *71*, 3–8.

(4) Upadhyay, A.; Kanika, N.; Mandhar, Y.; Batabyal, M.; Raju, S.; Jaiswal, S.; Butcher, R. J.; Kumar, S. Synthesis of Cobalt(Ii) Phenolate Selenoether Complexes to Mimic Hydrogenase-like Activity for Hydrogen Gas Production. *Dalt. Trans.* **2022**, *52*, 159–174.

(5) Batabyal, M.; Jaiswal, S.; Jha, R. K.; Kumar, S. Directing Group Strategy for the Isolation of Organoselenium(VI) Benzoselenonates: Metal-Free Catalysts for Hydrogen Evolution Reaction. *J. Am. Chem. Soc.* **2024**, *146*, 57–61.

(6) Yadav, P.; Khoury, S.; Fridman, N.; Sharma, V. K.; Kumar, A.; Majdoub, M.; Kumar, A.; Diskin-Posner, Y.; Mahammed, A.; Gross, Z. Trifluoromethyl Hydrolysis En Route

to Corroles with Increased Druglikeness. *Angew. Chemie* **2021**, *133*, 12939–12944.

(7) Mahammed, A.; Giladi, I.; Goldberg, I.; Gross, Z. Synthesis and Structural Characterization of a Novel Covalently-Bound Corrole Dimer. *Chem. Eur. J.* **2001**, *7*, 4259–4265.

(8) Sudhakar, K.; Mahammed, A.; Fridman, N.; Gross, Z. Iodinated Cobalt Corroles. J. Porphyr. Phthalocyanines 2017, 21, 900–907.

(9) Ganguly, S.; Conradie, J.; Bendix, J.; Gagnon, K. J.; McCormick, L. J.; Ghosh, A. Electronic Structure of Cobalt-Corrole-Pyridine Complexes: Noninnocent Five-Coordinate Co(II) Corrole-Radical States. *J. Phys. Chem. A* **2017**, *121*, 9589–9598.

(10) Jacques, P. A.; Artero, V.; Pécautb, J.; Fontecave, M. Cobalt and Nickel Diimine-Dioxime Complexes as Molecular Electrocatalysts for Hydrogen Evolution with Low Overvoltages. *Proc. Natl. Acad. Sci.* **2009**, 106, 20627–20632.

(11) G. Yang, J. H. Cen, J. Lan, M. Y. Li, X. Zhan, G. Q. Yuan, H. Y. Liu, ChemSusChem 2022, 15, e202201553.

(12) Y.-Q. Zhong, M. S. Hossain, Y. Chen, Q.-H. Fan, S.-Z. Zhan, H.-Y. Liu, Transition Metal Chemistry 2019, 44, 399-406.

(13) Lv Z.Y., Yang G., Ren B.P., Liu z.y., Zhang H., Si L.P., Liu H.Y., and Chi-Kwong Chang C.K; Electrocatalytic Hydrogen Evolution of the Cobalt TriarylCorroles Bearing Hydroxyl Groups. *Eur. J. Inorg. Chem.* **2023**, 26, e202200755.

(14) B. Wan, F. Cheng, J. Lan, Y. Zhao, G. Yang, Y.-M. Sun, L.-P. Si, H.-Y. Liu, *Int. J. of Hydrogen Energy,* **2023**, 48, 5506-5517.

(15) W.-Y. Peng, J. Lan, Z.-M. Zhu, L.-P. Si, H. Zhang, S.-Z. Zhan, H.-Y. Liu, *Inorg. Chem. Comm.,* **2022**, 140, 109453.

(17) H. Chen, D.-L. Huang, M. S. Hossain, G.-T. Luo, H.-Y. Liu, *J. of Coord. Chem.* **2019**, 72, 2791-2803.

(18) B. Mondal, K. Sengupta, A. Rana, A. Mahammed, M. Botoshansky, S. G. Dey, Z. Gross, A. Dey, *Inorg Chem* **2013**, 52, 3381-3387.
